# Ionic Rigid Organic Dual-State Emission Compound With Rod-Shaped and Conjugated Structure for Sensitive Al^3+^ Detection

**DOI:** 10.3389/fchem.2022.807088

**Published:** 2022-03-07

**Authors:** Hongying Lv, Lingzhong Wei, Song Guo, Xiaofeng Zhang, Feixia Chen, Xiaojin Qin, Chun Wei, Bingli Jiang, Yongyang Gong

**Affiliations:** ^1^ College of Pharmacy, Guilin Medical University, Guilin, China; ^2^ Key Laboratory of New Processing Technology for Nonferrous Metal and Materials, Ministry of Education, Guangxi Key Laboratory of Optical and Electronic Materials and Devices, College of Materials Science and Engineering, Guilin University of Technology, Guilin, China

**Keywords:** dual-state emission, Al^3^^+^ detection, organic conjugated compound, rod-shaped structure, mechanochromism

## Abstract

Dual-state emission (DSE) luminogens, a type of luminescent material which can effectively emit light in both dilute solution and solid states, have attracted tremendous attention, due to their widespread applications in chemical sensing, biological imaging, organic electronic devices, and so on. They overcome the shortcomings of aggregation-induced emission (AIE)-type compounds that do not emit light in dilute solutions and aggregation-caused quenching (ACQ)-type compounds that do not emit light in a concentrated or aggregated state. This work reports a novel ionic DSE material based on rigid rod-shaped organic conjugated structure using 4,4′-bis(2-sulfonatostyryl) biphenyl disodium salt (BSBDS); the ion repulsion effect can reduce the strong π–π interaction in aggregation and achieve high-efficiency luminescence in solution and solid states. In addition to excellent DSE characteristics, BSBDS also exhibits a mechanochromic nature and sensitive detection performance for aluminum ion (Al^3+^).

## Introduction

Organic luminescent materials have been widely used in various areas, such as organic light-emitting diodes (OLEDs) and chemical sensors (Reineke et al., 2009; Uoyama et al., 2012; Zhang et al., 2014; Meng et al., 2021). Traditional organic luminescent materials usually have large planar conjugated units, allowing them to exhibit bright emission in solution states; however, these materials often encounter the infamous aggregation-caused quenching (ACQ) effect and exhibit no or weak luminescence in the aggregated state, which extremely limits their wider applications. Fortunately, in 2001, Tang et al. discovered an opposite and interesting aggregation-induced emission (AIE) phenomenon (Luo et al., 2001), a group of non-luminescent molecules with a propeller-like conformation showed no emission in the solution state but strong emissions in the aggregation state. Then, restriction of the intramolecular motion (RIM) was proposed to well explain this phenomenon (Wang et al., 2015). The discovery of the AIE phenomenon has opened up a new robust method to produce novel organic luminescent materials with high-efficiency emissions in aggregated states.

In fact, an organic luminescent material that can emit light efficiently in both the solution and solid states will be an ideal luminogen, which combines the advantages of AIE and ACQ compounds and shows a broad application. In the past few years, organic dual-state emission (DSE) materials that can effectively emit light in both the solution and solid states have been reported sporadically and applied in OLEDs, cell imaging, and other fields. For instance, Tang et al. reported conjugation-induced rigidity and through-space conjugation in twisting molecules for designing novel DSE materials (Chen G et al., 2015; Chen L et al., 2015). Dong et al. reported a series of DSE compounds based on triphenylpyrrole (TPP) (Li et al., 2019) and triphenylquinoline (TPQ) (Dai et al., 2019). Goel et al. reported 5,6-dihydro-2h-pyrano [3,2-g]indolizine derivatives that exhibited DSE characteristics (Raghuvanshi et al., 2017). Ulrich et al. reported π-extended salicylaldehyde fluorophores showing a DSE nature (Stoerkler et al., 2021). Recently, a rational molecular design strategy has been presented based on triphenylamine *via* theoretical calculation and practical verification by the Gong group (Zou et al., 2022). However, although many efforts including various physical and chemical methods have been contributed to designing and exploring new DSE compounds, the research of DSE luminogens remains preliminary with relatively limited examples in the literatures (Belmonte-Vázquez et al., 2021). There still exists a serious lack of a universal molecular design strategy for DSE luminogens.

**FIGURE 1:**
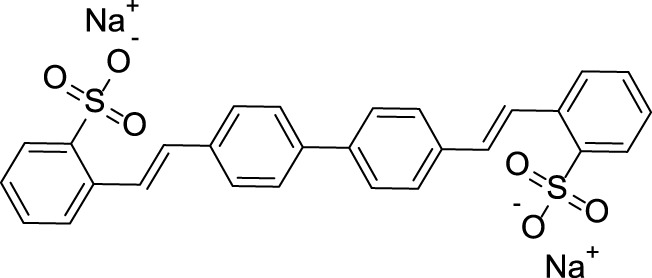
Chemical structure of BSBDS.

Herein, an ionic organic conjugated compound with a rigid rod shape was proposed to construct DSE luminogens. First, the rod-shaped structure can effectively inhibit the strong π–π interaction leading to the quenching of luminescence. Second, the ionic compound can avoid the formation of excimer and complex in the solution due to its own electrostatic repulsion. Combining the rod-shaped structure and ionic characteristics, novel DSE luminogens can be designed effectively to meet various application requirements. In our work, a typical fluorescent whitening agent named disodium 4,4′-bis (2-sulfonatostyryl) biphenyl (BSBDS) is used to verify our design strategy (Figure 1). Surprisingly, in addition to the DSE characteristics, BSBDS shows mechanochromic characteristics under force stimulation and can also be used for the identification and detection of aluminum ions (Al^3+^) in solution states.

## Experimental

### Reagents

4,4-bis(2-sulfonatostyryl) biphenyl disodium salt (C_28_H_20_Na_2_O_6_S_2_) was purchased from Tokyo Chemical Industry Co., Ltd. Methanol, anhydrous ethanol, and metallic salts were purchased from Xilong Scientific Co., Ltd. Tetrahydrofuran was obtained from Guangdong Guanghua Technology. Standard stock solutions of metal ions (Li^+^, K^+^, Ag^+^, Cu^+^, Ca^2+^, Mg^2+^, Zn^2+^, Cd^2+^, Co^2+^, Ni^2+^, Cu^2+^, Fe^2+^, Fe^3+^, and Al^3+^) were prepared with ultrapure water from the respective metallic salts (LiCl, KCl, AgNO_3_, CaCl_2_, MgCl_2_, AlCl_3_ 6H_2_O, Cu(NO_3_)_2_ 3H_2_O, FeCl_2_ 4H_2_O, FeCl_3_ 6H_2_O, Zn(NO_3_)_2_ 6H_2_O, CuCl, concentrated sulfuric acid (H_2_SO_4_), CdCl_2_ 5/2H_2_O, NiCl_2_ 6H_2_O, CoCl_2_ 6H_2_O, and K_2_CO_3_). All the reagents were of the analytical reagent grade and used as received. All the aqueous solutions were prepared with distilled water supplied from a Milli-Q purication water system. Tetrahydrofuran (THF) was distilled from sodium/benzophenone under nitrogen before use.

### Instrumentations

Ultraviolet–visible (UV-Vis) spectra were recorded on a UV3600 spectrophotometer. Fluorescence emission spectra and the quantum efficiency analysis were performed on a Horiba FluorMax-4 spectrofluorometer. X-ray diffraction (XRD) was measured on a X'Pert Pro X-ray diffractometer. The fluorescence lifetime was obtained using a Horiba QM-8000 fluorescence spectrophotometer. The luminescence quantum efficiency in solutions was measured using quinine sulfate as the standard solution (Φ_F_ = 54%, dilute sulfuric acid as the solvent). The size distribution of the suspensions for BSBDS in 90/10 THF–water was determined on a Malvern Zetasizer nano ZS90 instrument at 25°C. The toxicity of cells was confirmed with a Tecan Infinite F50 Microplate Reader. The fluorescence images of cells were gained on an Olympus FV 3000 laser scanning confocal microscope (LSCM).

### Theoretical Calculation

Geometry optimizations and frequency calculations were performed by using the density functional theory (DFT), and the time-dependent density functional theory (TD-DFT) was applied to study the low-lying excited states with the B3LYP functional and 6-31+G(d) basis set. All above calculations were carried out with the Gaussian 16 B.01 package (Frisch et al., 2016). Gaussian calculation results were analyzed by Multiwfn (Lu and Chen, 2012) and VMD (Humphrey et al., 1996) software.

### MTT Assay for Cytotoxicity Evaluation

The toxicity of BSBDS was evaluated using conventional MTT assay. HeLa cells were seeded in a 96-well plate at a density of 1 × 10^4^ cells per well in DMEM. After 24 h of growth, the medium in the cells was replaced with a fresh solution (100 µL) containing BSBDS with different concentrations ranging from 12 (control) to 96 µM. Following the incubation of cells with the BSBDS for 24 h, the medium was replaced with a fresh medium containing MTT (0.5 mg/ml) and incubated for another 4 h. The medium and MTT solution were replaced with 150 µL DMSO in each well. The plate was shaken well and the absorbance at 492 nm was recorded using a microplate reader (Infinite F50, Tecan). The cell viability was calculated as the ratio of average absorbance intensity of samples to that of the control.

### Cell Imaging

HeLa cells were seeded and grown in DMEM supplemented by 10% fetal bovine serum (FBS) and 1% penicillin/streptomycin (PS) under ambient conditions of 5% CO_2_ and humidity at 37°C. At 80% confluence, these cells were trypsinized, and about 2 × 10^5^ cells were added to each well of a 6-well culture plate. After 24 h of growth, the cells were incubated with a commercial dye (Nuclear Red™ LCS1) at 37°C for 20–30 min; then, Al^3+^ and BSBDS were added to the culture medium for incubating (washing off the excess agent and washing cells with PBS three times before adding different reagents). Finally, the cells were fixed with 4% paraformaldehyde. HeLa cell imaging was carried out by using an LSCM (OLYMPUS, FV3000).

## Results and Discussion

### Photophysical Properties of BSBDS in Solutions

**FIGURE 2:**
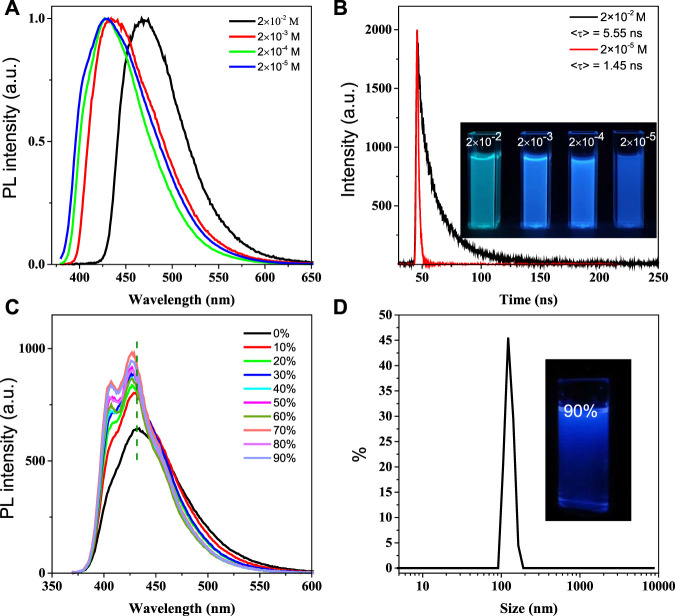
Emission spectra of BSBDS at different concentrations **(A)**; time-resolved emission decay curves patterns of 2 × 10^−2^ M and 2 × 10^−5^ M, and luminescence photographs of solutions at different concentrations under excitation of 365-nm ultraviolet light **(B)**; emission spectra of BSBDS in water and THF–water mixtures, concentration = 2 × 10^−5^ M **(C)** and size distribution and photograph of BSBDS suspensions in 90/10 THF–water mixture **(D)**; excitation wavelength **(A, C)** = 360 nm, slit width = 2 nm.

In view of good water solubility of BSBDS, the photophysical properties of BSBDS were measured in water. The absorption spectrum of BSBDS shown in Supplementary Figure S1 exhibits the maximum absorption peak at 350 nm. The emission spectra and time-resolved emission decay curves of BSBDS in aqueous solution at different concentrations are shown in Figures 2A,B, and the results suggest that BSBDS solution exhibited bright a blue to green emission from low to high concentrations. When the concentration is less than or equal to 2 × 10^−4^ M, the emission peak of BSBDS was at 430 nm. When the concentration reaches 2 × 10^−2^ M, the emission wavelength significantly red-shifted to 470 nm, which can be attributed to the formation of local excimers in BSBDS at high concentrations. The fluorescence lifetime/quantum efficiency of BSBDS at the concentration of 2 × 10^−2^ and 2 × 10^−5^ M are 5.55 ns/62.3% and 1.45 ns/74.3%, respectively, which indicates that luminescence at low concentration (≤2 × 10^−4^ M) originates from the intrinsic monomer emission of BSBDS, and luminescence at high concentration (2 × 10^−2^ M) is derived from excimer emission of BSBDS. It is worth noting that the fluorescence intensity of solution at the concentration of 2 × 10^−5^ M is greater than those of at 2 × 10^−2^ ∼ 2 × 10^−4^ M (Supplementary Figure S2), but the opposite is observed on the luminescence photos (Figure 2B). According to previous studies, this phenomenon can be attributed to the self-absorption effect. This phenomenon was reported in Henderson et al. (1977)and Yamasaki et al. (1997). When the excited light irradiates the sample at very high concentrations, there exists an overlap between the absorption spectrum and emission spectrum, and thus some emitted photons are absorbed by other solute molecules in the solution before reaching the detectors.

To investigate the photophysical properties of BSBDS at the aggregate state, the emission spectrum of BSBDS in THF/water mixed solution was further tested (H_2_O and THF are good and poor solvents of BSBDS, respectively) as shown in Figures 2C,D. As the content of THF increases (volume fraction), the luminescent intensity of BSBDS in the THF/water mixed solution shows a significant increase, while the emission peak exhibits a slight blue-shift from 430 to 427 nm. This may be due to the formation of a unique nanostructure from a single-molecule state as the THF content increases. Then, the measurement of particle size for BSBDS in the solution with a THF content of 90% was carried out, and the results indicated that nanoparticles with an average particle size of 119 nm were formed (Figure 2D). It is worth noting that the rod-shaped rigid unit in the middle of BSBDS is lipophilic, and the two ends are hydrophilic sodium sulfonate; this amphiphilic compound can form a variety of unique nanostructures. Compared with the photophysical properties of solution at the concentration of 2 × 10^−2^ M, the solution with the THF content of 90% should have no excimer formed. The slight blue-shift of the spectrum with the increasing THF content is difficult to explain because there is no specific molecular packing model in the nanoparticles. In addition, the increase of luminescent intensity with the increasing THF content can be attributed to RIM in nanoparticles, which is similar to the AIE mechanism (Wang et al., 2015).

### Photophysical Properties of BSBDS in the Solid State

**FIGURE 3:**
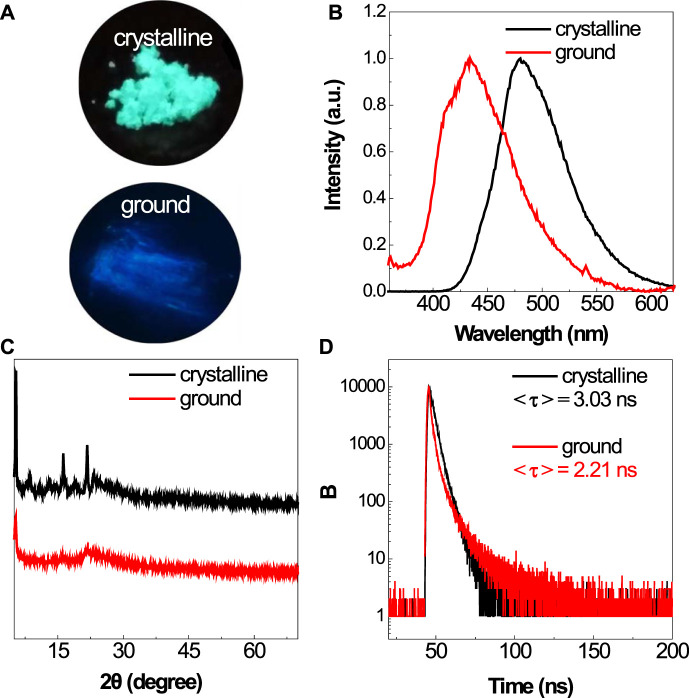
Photographs of the crystalline and ground powders of BSBDS taken under 365-nm UV light irradiation **(A)**. PL spectra **(B)**, XRD **(C)**, and time-resolved emission decay curves **(D)** of the crystalline and ground powders of BSBDS.

Similar to their solution, the BSBDS powder shows bright green light upon UV illumination (Figure 3A). According to our previous experience (He et al., 2019), the rod-like ion compound with highly efficient emission in the solid state may possess mechanochromic characteristics. Then, the response of BSBDS to force stimulation was carried out carefully as shown in Figures 3A,B. The crystalline powder of BSBDS recrystallized from water/methanol showed bright green emission peaking at 480 nm; however, after being ground gently with a spatula on filter paper, they exhibited a large blue shift with a deep blue luminescence peaking at 433 nm. In order to explore the mechanochromic mechanism, the XRD measurement of solid samples before and after grinding was performed. As shown in Figure 3C, the XRD pattern of the crystalline BSBDS powder exhibited sharp and intense diffraction peaks, which indicated their well-defined microcrystalline lattices. However, their grounded samples did not show any noticeable reflections but had broad diffused halos with low intensity, suggesting their disordered amorphous nature. Clearly, the transition between the ordered crystalline and disordered amorphous states is crucial to the mechanochromism. Besides the change in emission color, the photoluminescence (PL) efficiency of the powders after being ground also increased considerably from 65.5 to 84.5%, which might be ascribed to the destruction of π–π interaction between adjacent molecules in crystalline solid states. Normally, an increased emission efficiency would result in a prolonged lifetime, whereas the formation of excimer would decrease the lifetime. Here, the <τ> values of the crystalline powders and ground solids of DBSDB were determined as 3.03 and 2.21 ns (Figure 3D), respectively. The increased efficiency along with the decreased lifetime of the ground samples verified that the excimer was locally formed in the rigid element, and it was destroyed upon mechanical stimuli.

### Theoretical Calculation

**FIGURE 4:**
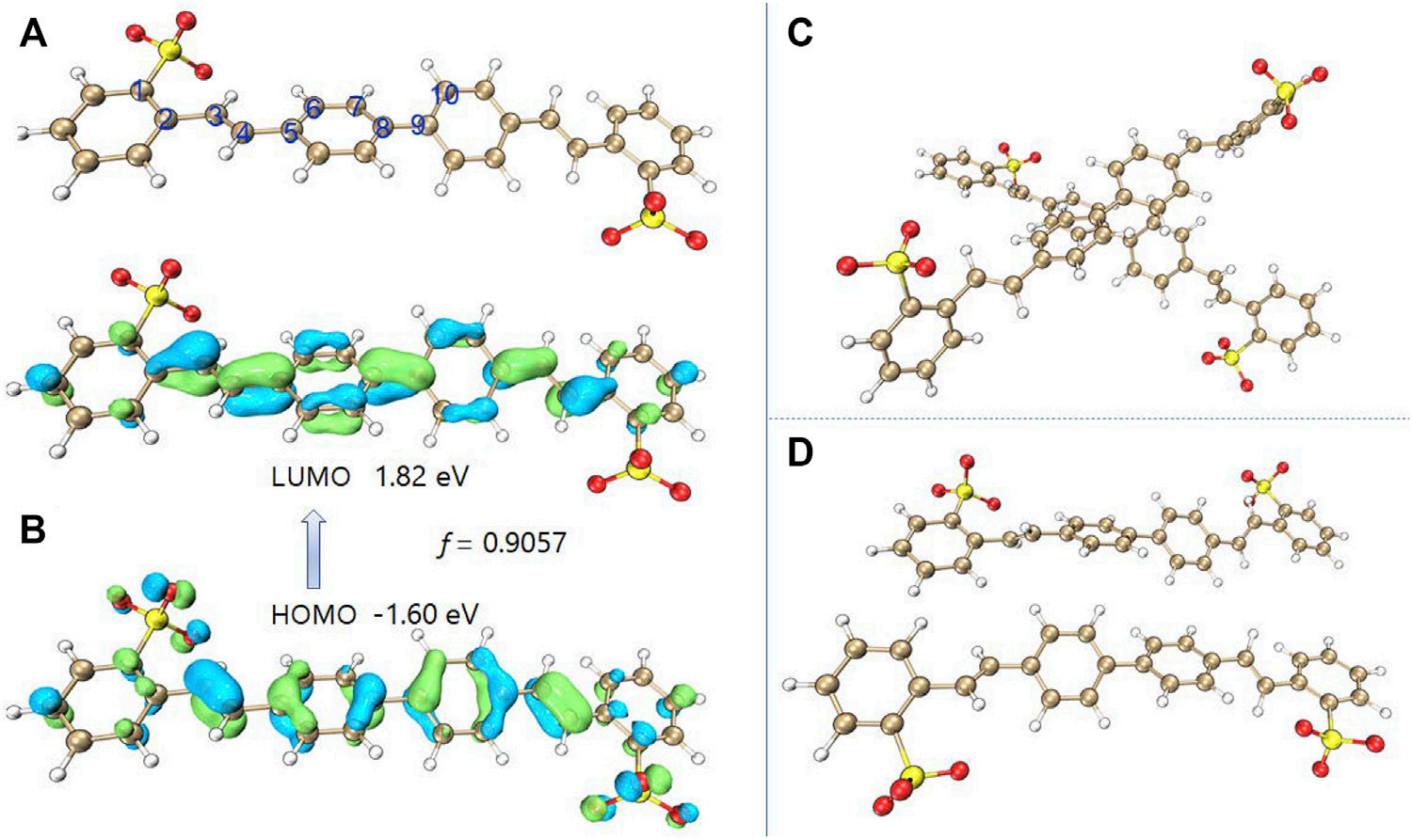
Molecular of the optimized BSBDS anion **(A)**, LUMO and HOMO distribution of BSBDS **(B)**, and some possible dimer configurations **(C,D)**.

In order to deeply understand the mechanism of DSE, the density functional theory and time-dependent density functional theory were applied to study the mechanism of luminescence. First, the B3LYP/6-31 + g(d) method was used to optimize the molecular structure of BSBDS. It is worth noting that the sodium ions (Na^+^) in the solution had no effect on the luminescence of the BSBDS, so the optimized structure did not contain the content of Na^+^. The optimized anion structure of BSBDS is shown in Figure 4A. BSBDS presents a typical rod-like structure, and the dihedral angles of ∠C1-C2-C3-C4, ∠C3-C4-C5-C6, and ∠C7-C8-C9-C10 are 19.04, 2.78, and 35.03°, respectively. A smaller dihedral angle means that BSBDS has a larger degree of conjugation. The lowest unoccupied molecular orbital (LUMO) and highest occupied molecular orbital (HOMO) of BSBDS distributed on the whole molecule, and the large degree of overlap indicates that they have no internal charge transfer (ICT) effect (Figure 4B). It coincides with the basically unchanged spectral position in the THF–water mixed solution with a different THF content. In addition, the transition process from HOMO to LUMO has a larger oscillator strength (*f* = 0.9057). *f* has a certain relationship with the luminous efficiency (Hilborn, 1982); if the *f* is less than 0.01, it indicates that the transition is forbidden. Since BSBDS is an amphiphilic compound, it has many ways of molecular accumulation in aggregates or high concentrations. However, due to the rod-like structure and ion repulsion effect, it is possible for BSBDS to form excimers in local areas (Figures 4C,D) to make its luminescence red-shifted, and the luminous efficiency is reduced to a small extent.

### Metal Cation Detection

**FIGURE 5:**
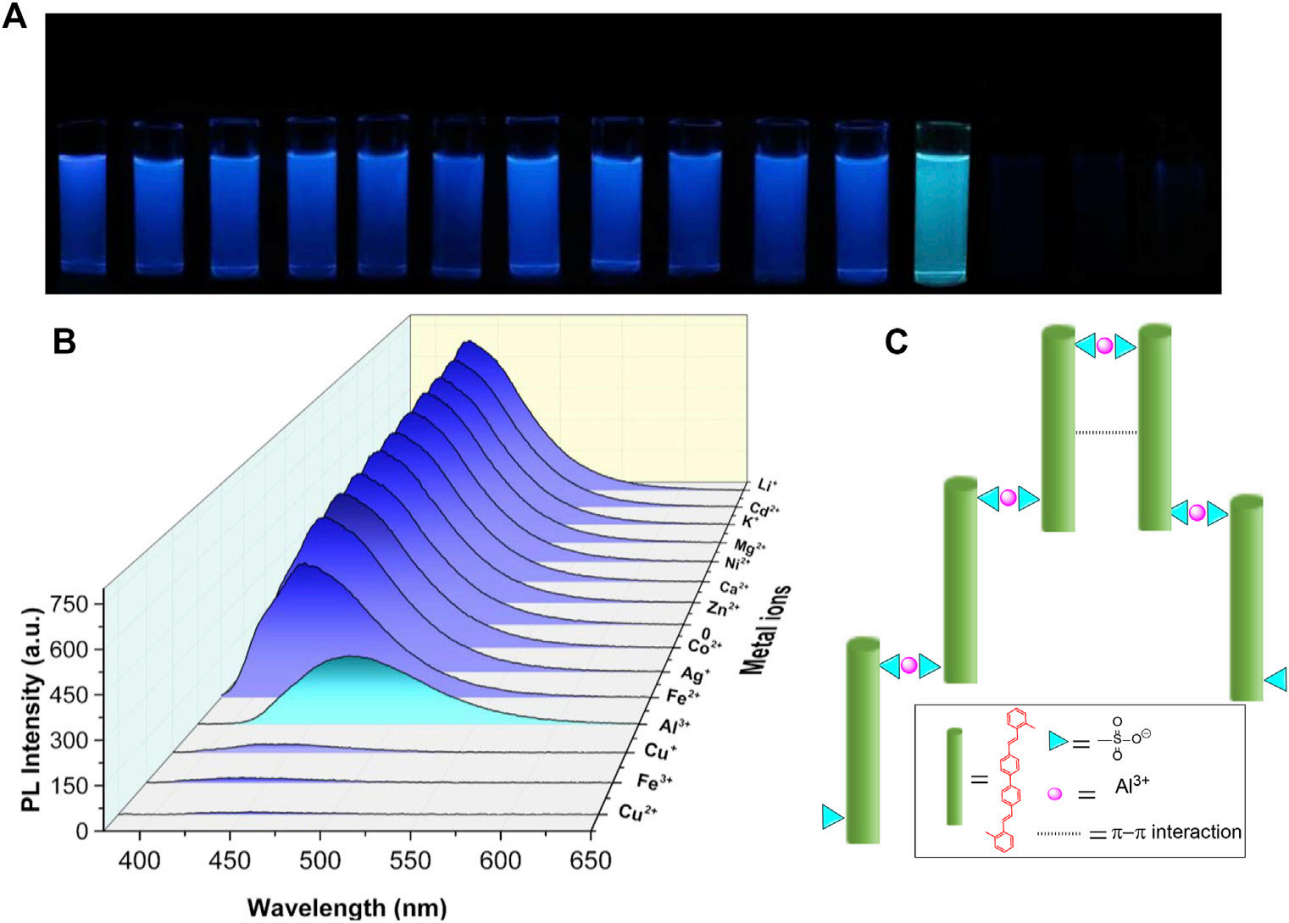
Photographs **(A)** and PL spectra **(B)** of mixed solution of BSBDS and different metal ions, and the schematic diagram of the mechanism of BSBDS detecting Al^3+^ **(C)**.

As mentioned previously, the photophysical properties of BSBDS can significantly change after forming an excimer or a complex at a high concentration. Therefore, it is assumed that BSBDS in the dilute solution state can form an excimer or exciplex after binding with a certain cation, which significantly changes its photophysical properties, and it can maybe be used to detect certain ions in the aqueous solution. In order to verify this hypothesis, 14 kinds of metal cations, Li^+^, Ca^2+^, Zn^2+^, K^+^, Mg^2+^, Fe^2+^, Ag^+^, Cd^2+^, Co^2+^, Cu^2+^, Cu^+^, Ni^2+^, Al^3+^, and Fe^3+^ were prepared. First, 2.0 ml of different metal cation solutions with a concentration of 2.0 × 10^−3^ M were added to 3.0 ml of BSBDS aqueous solution with a concentration of 2.0 × 10^−5^ M. Surprisingly, as shown in Figure 5A, the luminescent color of BSBDS changed instantly after the addition of Al^3+^ under 365-nm UV light irradiation. In addition, when Fe^3+^, Cu^2+^, and Cu^+^ ions were added to the BSBDS solution, the luminescence of the mixed solution quenched due to the electron or energy transfer. Furthermore, PL spectra of mixture solutions of BSBDS combined with different metal ions were studied (Figure 5B). Except for the above Cu^+^, Cu^2+^, Fe^3+^ which led to quench the emission of BSBDS, the addition of other ions showed a little effect on the emission peak (∼430 nm) and intensity. However, when Al^3+^ ions were added to the BSBDS solution, the emission peak red-shifted from 430 to 475 nm, which was consistent with the emission behavior at the concentration of 2 × 10^−2^ in the solution and crystalline powder. The results indicated that BSBDS complexed with Al^3+^ formed an excimer (Figure 5C). Furthermore, we had further studied the detection performance of BSBDS in the solid state for aluminum ions. First, the blank filter paper is soaked in a 2 × 10^−5^ M BSBDS aqueous solution for 1 min and then dried. The solution containing different metal ions was filtered through a filter paper containing BSBDS. After drying, it is found that BSBDS can still detect metallic aluminum ions (Supplementary Figure S3).

**FIGURE 6:**
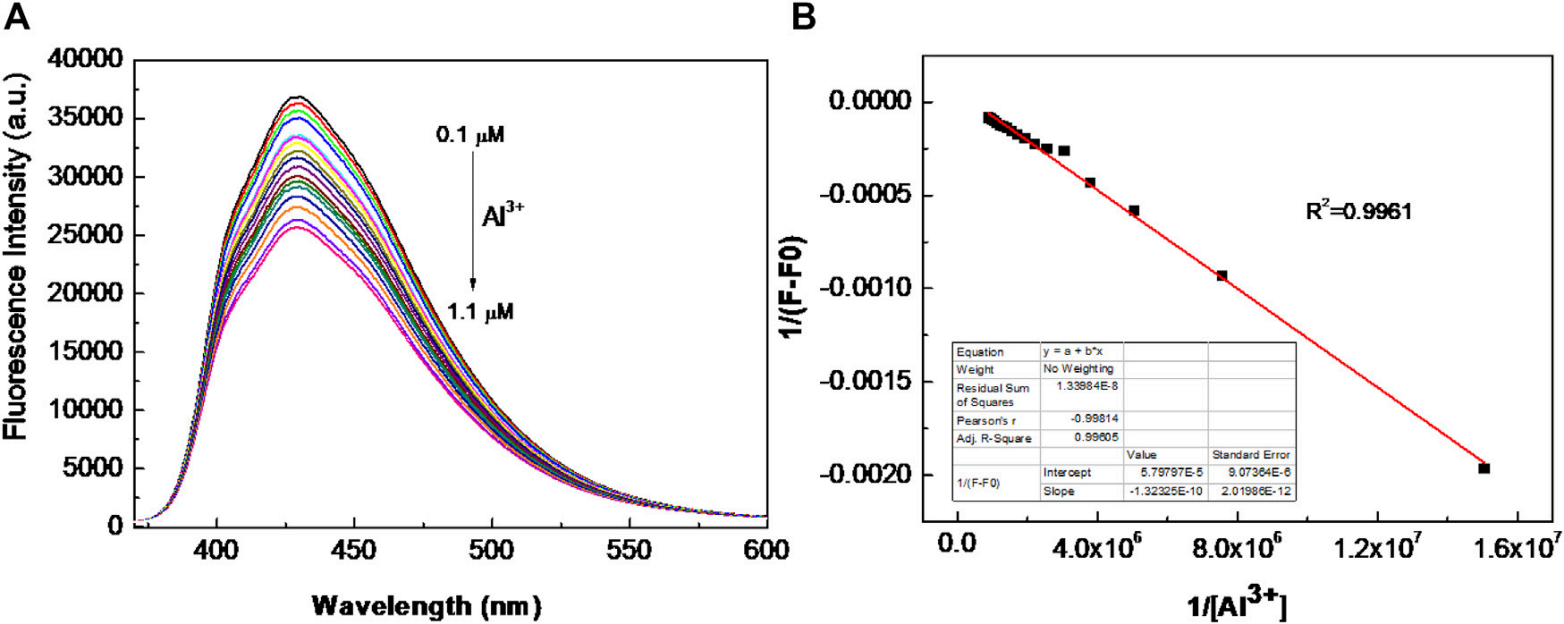
**(A)** Fluorescence intensity vs. Al^3+^ ion concentration in the range of 0.1–1.1 μM; and **(B)** the Benesi–Hildebrand plot of BSBDS with Al^3+^ (F: fluorescence emission intensity of BSBDS for given Al^3+^ concentration and F_0_: fluorescence intensity in absence of Al^3+^).

Furthermore, the sensitivity is also an important factor for a chemical sensor. Then, different amounts of Al^3+^ (10 μL) were added sequentially to the BSBDS aqueous solution to estimate the detection limit of Al^3+^. Upon the addition of Al^3+^, the fluorescence intensity of BSBDS at around 430 nm gradually declined due to electron or energy transfer (Haldar et al., 2016) (Figure 6A and Supplementary Figure S4). Based on the results, a good linear curve was obtained in the range of 0.1–1.1 μM (Figure 6B), and the limit of detection (LOD) was found to be 2.09 × 10^−8^ M according to computational method of 3σ/k (Wang et al., 2008) (k are standard deviation of intercept and slope of the linear curve, respectively). It is worth noting that the detection limits for Al^3+^ in drinking water are 7.4 × 10^−6^ M according to the World Health Organization (WHO) regulations (Dong et al., 2016). Therefore, BSBDS can be used as a convenient and sensitive fluorescent probe for the detection of Al^3+^ in drinking water. The dynamic quenching constant (K_sv_) is calculated *via* the Stern–Volmer formula (Dhingra et al., 2020) F_0_/F = 1+K_q_×τ_0_×[Q] = 1+Ksv×[Q], where K_q_ is the dynamic quenching rate constant, τ_0_ is the average lifetime of the lumiongens, and [Q] is the concentration of Al^3+^. We got the function of y = 362113.11254x+0.99312, so the quenching constant in Supplementary Figure S5 is 362113 L/mol.

### Cell Imaging

**FIGURE 7:**
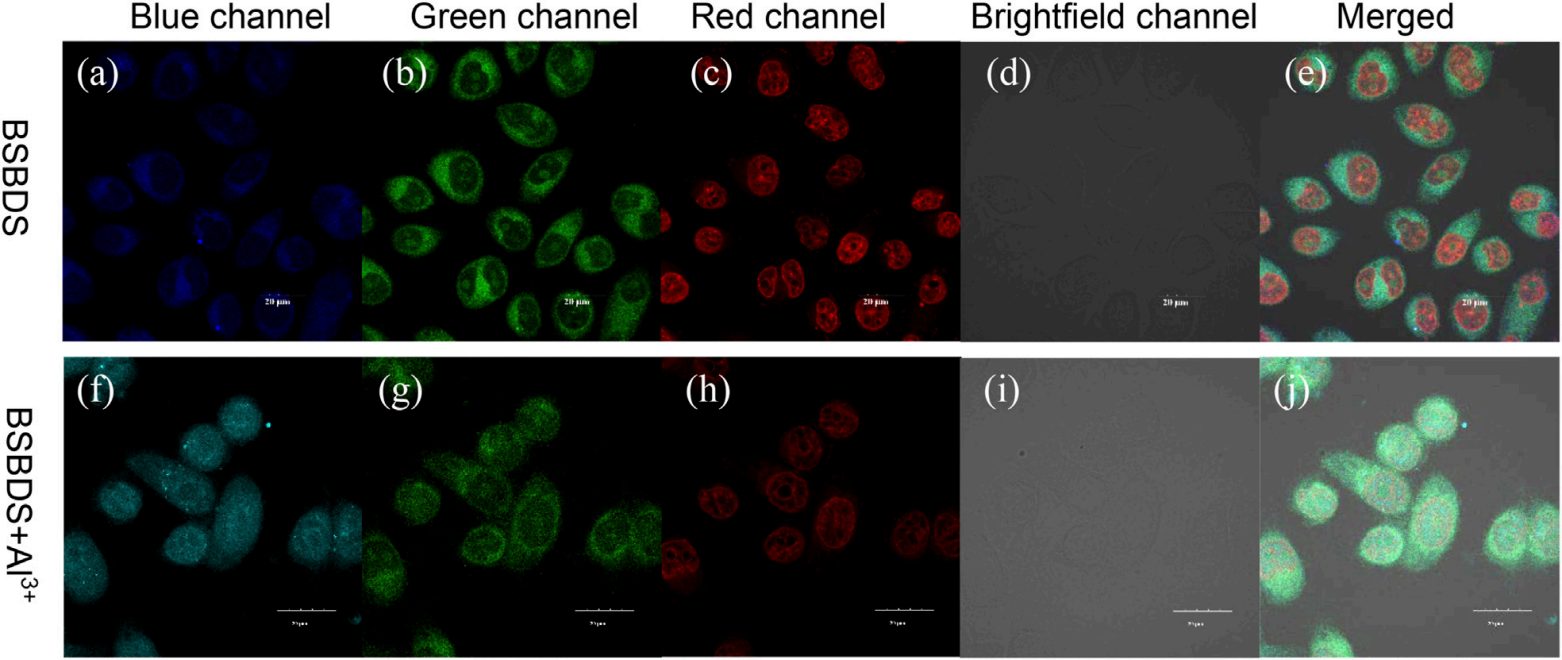
Confocal images of HeLa cancer cells incubated with BSBDS **(A–E)** and BSBDS + Al^3+^ **(F-J)** for 30 min **(A,F)**, **(B,G)**, **(C,H)**, **(D,I)**, and **(E,J)** are field, blue, green, red, bright, and merged channel images, respectively. Excitation the wavelength for blue, green, and red channels are 405, 455, and 488 nm, respectively. The length of the ruler in the picture is 20 μm.

In order to explore the biological feasibility of BSBDS, cytotoxicity of BSBDS was evaluated by MTT assay. As shown in Supplementary Figure S6, BSBDS was non-cytotoxic to living cells. It means that BSBDS has good biocompatibility, and it is safe for cell-imaging or biological applications. In view of the non-toxic nature of BSBDS, intracellular detection of Al^3+^ was performed. HeLa cells were incubated with Nuclear Red™ LCS1 (nucleus-tracker, a commercial dye by AAT Bioquest®, Inc.) for 30 min, and then treated with 20 μM BSBDS for another 1 h. The confocal images of HeLa cells showed a certain amount of a fluorescent signal in pseudo colors with blue and green from BSBDS, which mainly appeared in the cytoplasm of HeLa as displayed in Figures 7A,B, and the red signal of nucleus-tracker Nuclear Red™ LCS1 was indeed localized in the nucleus of HeLa cells; it was localized in different area with BSBDS (shown in Figures 7C–E). The other one is the experimental group which was preincubated with Al^3+^; it emitted intense blue fluorescence signal (Figure 7F) in comparison with Figure 7A; and weaker red fluorescence in the nucleus (Figure 7H) compared with the group preincubated without Al^3+^ (Figure 7C). The fluorescence signal can also be precisely observed in the nucleus in Figure 7H. We thus speculate that with the entry of Al^3+^ BSBDS emits strong light in both the cytoplasm and nucleus when excited by a 405-nm laser; Al^3+^ can also help BSBDS to enter into the nucleus. The results indicated that BSBDS is cell-permeable and could be used for dynamic imaging and Al^3+^ tracking in cells.

## Conclusion

In summary, we propose an ionic rod-shaped organic compound for the construction of high-efficiency DSE luminescent materials. Due to the large rod-shaped conjugation structure, BSDBS can emit light efficiently in the state of dilute solution. At the same time, the repulsive effect of ions at high concentrations can lead the rod-shaped molecules only form excimers locally, avoiding the aggregation quenching effect and achieving highly efficient luminescence in concentration and aggregation states. BSBDS was selected as a model compound to validate the molecular design strategy, and the results showed that the compound possessed high-fluorescence quantum yield in both solution and solid states. In addition, BSDBS has recognition ability for Al^3+^ in the solution state, obvious mechanochromism properties in the solid state, and good biocompatibility, which can be used for cell imaging. The unique chemical structure characteristics and excellent optical properties of this ionic rod-shaped organic compound make it exhibit broad applications in many fields, such as organic optoelectronics, chemical sensing, ion detection, and biological imaging. This work will provide new ideas for the development of novel DSE materials.

## Data Availability

The original contributions presented in the study are included in the article/Supplementary Material, further inquiries can be directed to the corresponding authors.
