# Supplementary material for: Ionic Rigid Organic Dual-State Emission Compound With Rod-Shaped and Conjugated Structure for Sensitive Al3+ Detection
Source: Front Chem. 2022 Mar 7;10:807088. doi: 10.3389/fchem.2022.807088 (PMC8940517; doi:10.3389/fchem.2022.807088)
Supplement: Supplementary file 1 [file DataSheet1.PDF]

Supplementary Material

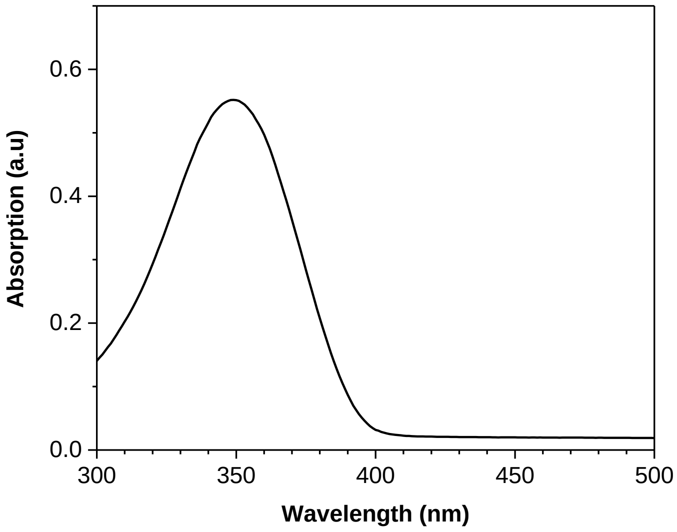

Supplementary Material Figure 1 Absorption of BSBDS in 2×10^{-5} M water.

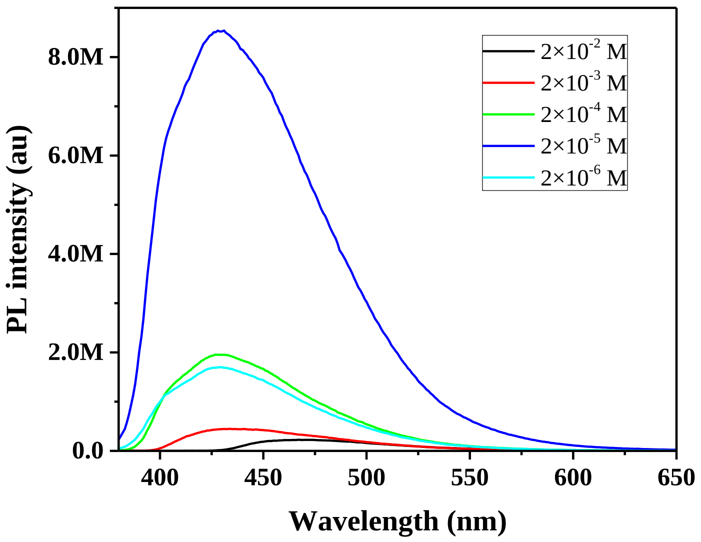

Supplementary Material Figure 2 Fluorescence intensity of BSBSD solutions with different concentrations.

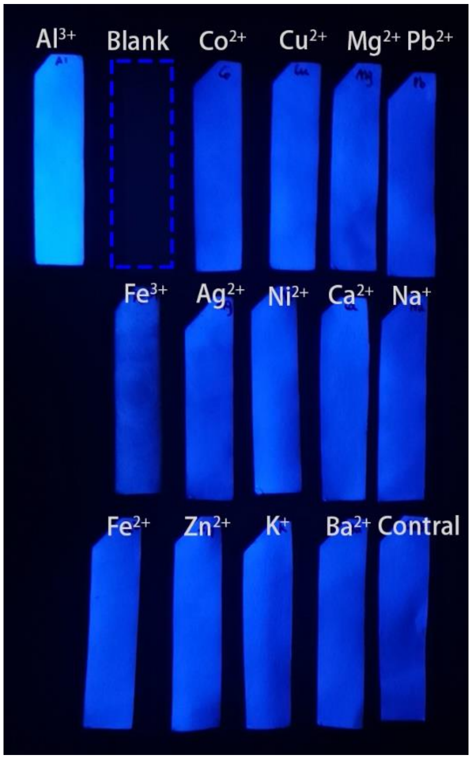

Supplementary Material Figure 3. Fluorescence photos of filter paper containing BSBDS for aluminum ion detection under 365 nm UV light.

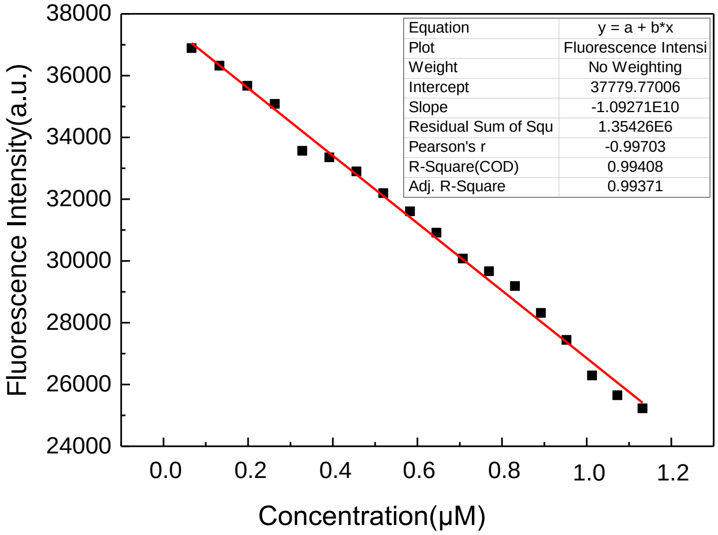

Supplementary Material Figure 4 Linear fit of the fluorescence intensity *vs* Al^{3+} ion concentration in the range of 0.1 - 1.1 μM;

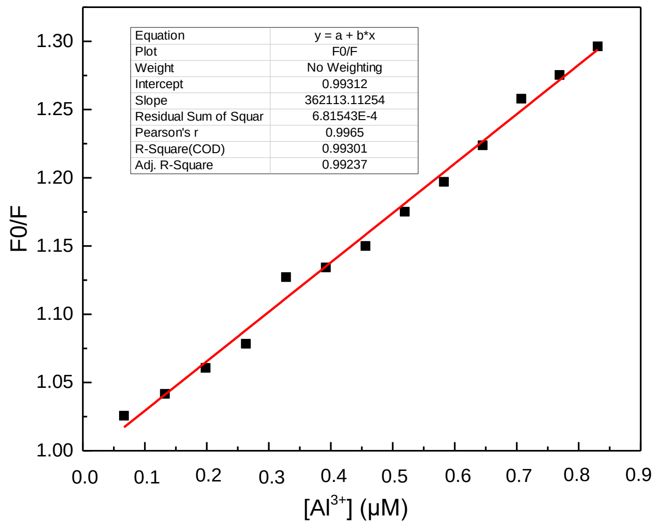

Supplementary Material Figure 5 Stern-Volmer plot of BSBDS in the presence of Al3+ with different concentrations (0-0.8 μM).

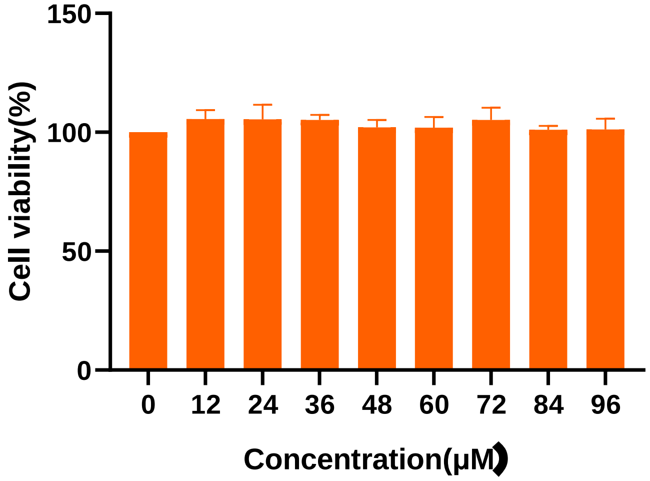

Supplementary Material Figure 6 Cytotoxicity on Hela cell by MTT assay in BSBDS different concentrations
